# Micronucleus Evaluation in Exfoliated Human Buccal Epithelium Cells among E-Waste Workers in Payatas, the Philippines

**DOI:** 10.5696/2156-9614-10.28.201213

**Published:** 2020-12-07

**Authors:** Julie S. Berame, Aris A. Lapada, Frosyl F. Miguel, Elisa C. Noguera, Zeba F. Alam

**Affiliations:** 1 Education/Biology Department, Caraga State University, Butuan City, Philippines; 2 Education Department, Eastern Samar State University, Borongan City, Philippines; 3 Science and Technology Department, Ramon Magsaysay High School, Manila, Philippines; 4 Science Department, Manuel Roxas High School, Manila, Philippines; 5 Biology Department, De La Salle University, Manila, Philippines

**Keywords:** micronuclei, exfoliated buccal epithelium cells, e-waste recyclers, genotoxicity

## Abstract

**Background.:**

The improper recycling of electronic waste (e-waste) by informal recyclers often leads to contamination of the environment. E-waste contains organic and inorganic compounds along with heavy metals and trace elements. These pollutants can have a negative effect on humans. Biomonitoring can provide information on the sources, amount, geographical distribution, and adverse health effects of contaminants.

**Objectives.:**

The present study aimed to assess risks to the health of informal e-waste recyclers in Payatas, the Philippines due to their exposure to e-waste toxicity by examining the presence of micronuclei in buccal epithelium cells.

**Methodology.:**

Frequencies of binucleated cells (BNc) and abnormal cells were obtained from the buccal epithelium of the study population composed of e-waste exposed recyclers (n=40) and a control group (n=52). Descriptive statistics and regression analysis were employed for the data analysis.

**Results.:**

Participants' gender, occupation, smoking status, alcohol consumption, and the number of karyolitic cells of both groups were significantly associated. Only occupation in e-waste recycling and length of e-waste exposure were significantly associated in terms of the number of abnormal cells and micronuclei. Similar trends were found in the linear regression analysis drawn from participants' length of e-waste exposure with a significance of R^2^= 7346, indicating that as the length of e-waste exposure increased, the number of micronuclei found in the participants' buccal epithelium cells increased as well.

**Conclusions.:**

Longer exposure to e-waste materials may induce genotoxic damage in human cells which is a serious concern, leading to adverse effects to human health.

**Competing Interests.:**

The authors declare no competing financial interests.

## Introduction

Management of e-waste has become a major challenge in the Philippines due to a steady increase in the volume of local e-wastes in the recent past.^1^ E-waste consists of a broad range of electrical and electronic products, including computers, mobile phones, televisions, refrigerators, ovens, vacuum cleaners, toasters, printers, faxes, fluorescent tubes and their components, such as printed circuit boards.^2^ The generation of large volumes of bulky e-wastes containing a variety of hazardous substances is a major problem and threatens the environment.^3^ Due to lack of proper guidelines and local government regulations, e-waste is dumped along with solid waste at sites close to human dwellings like Barangay Payatas in Manila, the Philippines as well as in India and Nigeria.^4^

Based on our observations in Payatas, genotoxic exposure is expected among informal e-waste recycling workers due to improper handling of e-waste materials in this area. Most e-waste recyclers are solely engaged in collecting and selling e-waste materials in junk shops after breaking them down into saleable components, as this is profitable. Some small e-waste shops buy these electronic wastes and use some of its parts for electronic gadget repairs. Large junk shops buy volumes of e-wastes and may sell them on to other companies to recycle these materials. These e-waste recyclers use potentially dangerous techniques and methods such as acids or open air burning to break down the components and this leads to exposure to genotoxins contained in the e-waste. Most of the e-waste recyclers live in small shanty dwellings in the same area as the e-waste recycling activities, thereby increasing their chances of exposure to harmful chemicals and toxins.

In addition, e-waste toxins that have carcinogenic and genotoxic effects from organic pollutants including brominated flame retardants, hexavalent chromium, polycyclic aromatic hydrocarbons and heavy metals like lead (Pb), mercury (Hg), arsenic (As), cadmium (Cd), selenium (Se), and aluminum (Al) disperse into the environment and contaminate the air, surface and groundwater, sediment, biota and soil.^7,8^

In contrast, municipal waste recyclers collect mixed garbage and dispose of it at allocated dumping sites for solid waste. This solid waste contains unconsolidated wastes from hospitals, factories, malls, clinics, restaurants, and other establishments that often do not engage in sorting out wastes. The waste may or may not contain hazardous chemicals or genotoxins, hence it would be hard to establish a cause and effect relationship for municipality workers compared to e-waste recyclers where the mode of exposure can be validated.

Several techniques like genotoxicity assays used to detect compounds that induce genetic damage have been employed in the clinical diagnosis of disease. Genetic assays like the micronucleus assay have gained widespread acceptance as indicators of DNA damage and carcinogenicity.^10^ The micronucleus assay has been one of the most popular indicators of DNA damage for over 30 years.^10^ It is sensitive, rapid, inexpensive, relatively less invasive, and has been widely used as a tool for the detection of mutagenic and genotoxic effects of chemicals in the environment and rapid biomonitoring of human health risks from exposure to genotoxins.^11,12,13,14^

In addition, micronuclei can be seen and identified using a simple light microscope. Micronuclei are chromosome fragments that lag during anaphase in nuclear division; moreover, this occurs due to common genetic aberrations, which are also considered an indicator of genetic damage.^15^ The test protocol of micronuclei using the buccal epithelium is well recognized in medical and professional studies as an effective and sensitive method to detect genotoxicity.^16,17^ Previous studies on micronucleus scoring for measuring DNA and genotoxicity have examined genotoxic chemicals from e-waste dumpsites.^18–21^ The results obtained by these methods may be useful for future studies and the development of novel therapeutic approaches to e-waste-related cases.^22^ Micronucleus investigation is used for genotoxicity screening because it is a well validated method for genotoxicity testing.^17^

The utilization of electronic products contributes significantly to the waste stream. In 2004, the United Nations Environmental Programme (UNEP) reported that e-waste is increasing by 4% every year.^23^ Presently, the Philippines have no data on the exact quantity of e-waste being generated. Many potentially hazardous substances can be found in e-wastes, including Se, Cd, As, Pb, Hg, lithium (Li), tin (Sn), copper (Cu), silicon (Si), carbon (C), iron (Fe), aluminum (Al), polybrominated biphenyls (PBBs) and polybrominated biphenyls (PBDEs).^24–26^ E-waste recycling can recover materials that remain useful and e-waste recyclers can recover up to 95% of the useful materials from a computer, for example.^4,27^

Recycling of e-waste materials in the Philippines is very common. There are a number of junk shops that buy and sell e-waste materials in Barangay Payatas, Quezon City. Some recyclers of plastic and metal wastes have shifted to collecting e-waste materials since they can earn more compared to selling metal and plastic waste. The methods of the present study could be used to evaluate genotoxicity in workers in other urban cities in the country who are exposed to e-waste chemicals.^11,13,14^

The present study aimed to assess the risk to the health of informal e-waste recyclers in Payatas, the Philippines due to their exposure to e-waste toxicity by examining the presence of micronuclei in buccal epithelium cells.

## Methods

The study site is a predominantly urban poor barangay in the northeastern district of Quezon City, which is within the outer vicinity of the city of Metropolitan Manila. It has been a disposal site for the city's electrical waste and electronic equipment for over two decades, known as the Smokey Mountain dumpsite since 1995. The population of Payatas is estimated at 130,000, including roughly 6,000 recyclers/ scavenger families who were initially relocated by the local government from various other slum settlements. This workforce consists of expert but under-compensated waste pickers, including women and children, who supply recyclable e-waste materials to established waste recovery and recycling businesses.^5,6^

**Figure 1:**
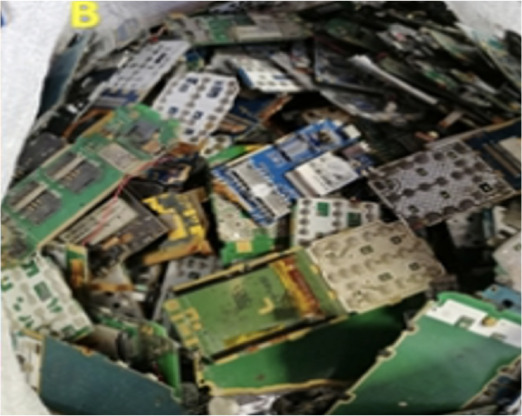
Examples of E-Waste Collected for Recycling

The present study was composed of three stages. The first stage involved the collection of demographic information on respondents such as age, sex, occupation, dietary habits, smoking, alcohol intake, and length of exposure to e-waste. The second stage involved the collection of buccal epithelial tissue from both the experimental and control group. The final stage involved processing samples at the Science and Technology Research Center (STRC), De La Salle University, Manila. A total of 40 adults aged from 18 to 70 years old who lived in Barangay Payatas, Quezon City in Metro Manila from birth up to present and had worked or were currently working in any jobs related to e-waste management like recycling computer spare parts *(Figure 1).*

The control group was comprised of 52 adults aged from 18 to 70 years old who were not e-waste recyclers. They lived outside the study area and came from different areas in Metro Manila and CALABARZON (Cavite, Laguna, Bulacan, Rizal, Quezon, all provinces immediately outside Metro Manila). The participants included tertiary students (18 years old and above), sale agents, workers in business offices or stores, and utility workers who resided away from the study area in Payatas in order to avoid study bias.

### Cell collection and staining

Each participant rinsed their mouth with distilled water and exfoliated buccal epithelium cells were collected through a swabbing inside the lining of the subjects' oral mucosa of both cheeks. The swabbed buccal mucosa cells were directly transferred to a conical tube with 0.9 saline solution; sealed, cool, and stored in the ice bucket with ice. Afterward, the conical tubes with buccal mucosa were brought to the Science and Technology Research Center (STRC) Laboratory at De La Salle University, Manila for staining and analysis.

### Micronucleus and nuclear abnormality testing in exfoliated buccal cells

Buccal epithelial tissues obtained from subjects were transferred into test tubes and were centrifuged for a span of 10 minutes at 5,000 rpm and the supernatant from the centrifuged samples were removed and substituted with 10 ml phosphate-buffered saline solution and centrifuged again for another 10 minutes at 5,000 rpm. The supernatants were pipetted out and transferred in 1.5 mL microcentrifuge tubes. Then 0.5 mL of a 3:1 fixative was added to the samples and centrifuged again for five minutes at 10,000 rpm. After this process, the pellet was smeared into clean slides and dried for 10 minutes. Slides were stained in 2% Giemsa solution for 5 minutes and washed with distilled water, air dried and then viewed in a binocular microscope.

**Figure 2:**
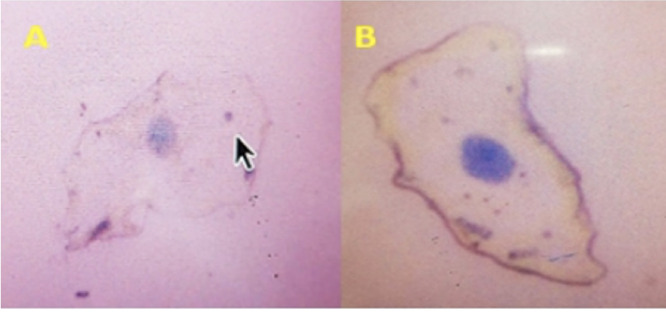
(a) Single micronucleus; (b) Multiple micronuclei

The micronuclei and NA were quantified using the scoring technique of Tolbert *et al.*, (2009)^11^ and a lab clicker was used to expedite this process. Normal cells with the nucleus are uniformly stained, oval or circle-shaped, and smaller than the cytoplasm. Micronucleated cells *(Figure 2a)* are characterized by the presence of the main nucleus and smaller structure denominated micronuclei. Micronuclei have a circle or oval shape with a length between 1/3 and 1/16 of the main nucleus; the intensity of the stain, texture, and plane is equal in both structures. Micronuclei characteristics include a rounded smooth perimeter suggestive of a membrane, less than a third the diameter of the associated nucleus, but large enough to discern shape and color; staining intensity similar to that of the nucleus; similar texture to that of the nucleus; same focal plane as a nucleus; absence of overlap with, or bridge to the nucleus; and a single cell may have multiple micronuclei *(Figure 2b)*.^13^

### Statistical analysis

Descriptive statistics were used to describe participants' age, length of e-waste exposure, smoking status, and drinking status. The micronuclei were counted and reported as the total number of occurrences in cells. The distributions of the variables were submitted for the normality test. To establish inferences, the chi-square test was used to determine the association of respondents' demographic profile and the number of micronuclei.

Moreover, the t-test for independent samples was used to test differences in age and the length of e-waste exposure between the experimental and control groups. Linear regression models were also drawn to compare the prevalence of micronuclei cells in the e-waste exposed group. A p-value of ≤ 0.05 was considered to be statistically significant. All statistical analyses were performed using the Statistical Package for the Social Sciences (SPSS) version 22.

## Results

Table 1 presents the demographic characteristics of the study participants. The data present information about the participants' exposure to e-wastes in Barangay Payatas in Quezon City, Manila. The present study is a pre-assessment of the complex nature of e-waste exposures to establish measures to educate e-waste workers on the effects of occupational exposures on their health. Descriptive statistics of t-test and the chi-square tests were used to describe and associate the two groups of samples.

**Table 1:** Demographic Characteristics of Study Participants by Group

**Variables**	**Control Group (N=52)**		**E-waste Group (N=40)**		**T-test p-value**

	Mean	SD	Mean	SD	
Age (yr)	34.5	9.24	34.25	12.34	0.47
Length of exposure (in years)	N/A	N/A	8.50	4.35	0.00
	N	%	N	%	Chi-square test *p-value*
Gender					
*Male*	35	67	31	78	0.02
*Female*	17	37	9	22	
Smoking					
*Yes*	4	8	31	78	0.00
*No*	48	92	9	22	
Alcohol consumption					
*Yes*	18	35	25	63	0.00
*No*	34	65	15	47	

Abbreviation: SD, standard deviation

As seen above, there was no significant difference in the age of participants (control and experimental) (*p=0.47*), but differences in length of exposure, occupation, smoking, and alcohol drinking were highly significant (*p=.000*). Those living near e-waste recycling sites or working in e-waste recycling had greater evidence of DNA damage than did those living in control towns.^39^

Among the four demographic profiles of the participants, three variables were highly significant, indicating that the two groups of exposed and unexposed participants differed in terms of their health conditions based on the findings of the present study. Table 2 shows the association between the demographic characteristics and total number of normal cells and micronuclei observed in the buccal epithelium of controls and e-waste recyclers in Barangay Payatas, Quezon City. The difference between the control and exposed group was significant in terms of increase in the number of micronuclei.

**Table 2:** Association Between Demographic Characteristics and Total Number of Normal Cells and Micronuclei Observed Among Participants

**Variables**	**Normal Cells**		**Micronucleus Cells**		**Chi-square *p*=value**

	N	%	N	%	
*Control*	51 724	65	630	16	0.00
*Exposed*	42 336	45	2 706	84	
Gender					
*Male*	66 441	71	2 875	86	0.70
*Female*	37 619	29	461	14	
Length of Exposure					
*<10 years*	26 365	62	1 801	80	0.02
*>10 years*	15 971	38	905	20	
Smoking					
*Yes*	41 241	44	2 353	71	0.31
*No*	51 660	66	983	29	

^*^p = 0.05

Table 3 presents the t-test for independent samples with respect to the number of micronuclei observed in the buccal epithelium of controls and e-waste recyclers. The standard deviations of both groups were statistically significant, with F=69.322, *p=0.00* in Levine's test for equality of variances, which means the two groups have the same variance of standard deviation of population. Moreover, there was a significant difference between the number of micronuclei in both groups with a t (90)=5.421, *p=0.00* in the t-test for independent samples. The results show that the e-waste workers had increased micronuclei compared to the control group.

**Table 3:** Independent T-test of the Number of Micronuclei Observed in the Buccal Epithelium of Control and E-waste Exposed Groups

**Group**	**Participants**	**Total Cells**	**No. of MN**	**F (Levine’s Test)**	**Sig**	**t**	**df**	**Sig**
Control	52	51 724	630	69.322	0.00	5.421	90	0.00
Exposed	40	42 336	2 706					

Abbreviations: df, degrees of freedom; MN, micronucles; Sig, significance

^*^ *p = 0.05*

Table 4 shows the chi-square test for abnormal cells in the buccal epithelium of control and e-waste recyclers with respect to binucleated and cells with karyolysis. The e-waste exposed group had a higher total number of abnormal cells (binucleated and karyolysis) compared to the control group. The chi-square test revealed a significant result for karyolysis (p=0.04). This suggests that the two groups do not have same variance of population with respect to the number of nuclear abnormalities.

**Table 4:** Test of Association Between the Number of Binucleated and Karyolitic Cells in the Control and Experimental Groups

**Variable**	**Control**		**E-waste**		**Chi-square**

	N	%	N	%	
Binucleated					
*Yes*	126	0.2	172	0.4	0.30
*No*	51724	99.8	42336	99.6	
Karyolysis					
*Yes*	361	0.7	866	2	0.04
*No*	51724	99.3	42336	98	

^*^ *p = 0.05*

The results suggest that e-waste workers are more prone to cellular abnormalities or cellular aberrations. In the present study, an increase in cellular damage was associated with an increase in the number of micronuclei. As the number of micronuclei increased, the number of cellular abnormalities likewise increased.

**Figure 3:**
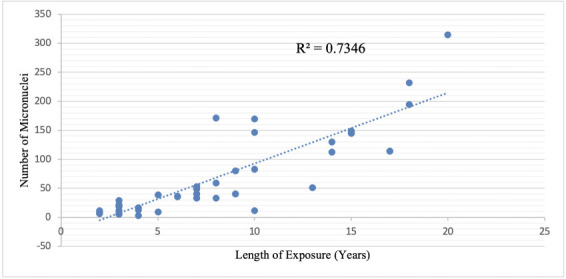
Linear regression analysis of the length of exposure of e-waste workers

Figure 3 illustrates that the length of exposure to e-waste can influence the number of micronuclei in a cell. An R^2^ = 0.7346 indicates that the length of exposure can influence the number of micronuclei of an individual exposed to e-waste. As the length of exposure increased, the number of micronuclei increased as well.

## Discussion

To the best of our knowledge, the present study is the first investigation to be published assessing evidence of the effects of exposure to e-waste among workers in the Philippines using buccal epithelium cells. The present study examined evidence of causality between exposure to e-waste and outcomes, focusing the strength and consistency of associations, plausibility, while considering alternative explanations.

E-waste practices may lead to potential adverse effects on humans, and e-waste management is a significant environmental health hazard.^29,30^ In Barangay Payatas, Quezon City, Manila, the majority of residents are involved in collecting and selling used electronic gadgets/appliances such as cell phones, TVs, radios, refrigerators, batteries, and other end-of-life materials. In areas where these e-waste activities take place, environmental harm is evident by the presence of unhealthy air coming from waste materials junked along the roads and dumped in warehouses, adversely affecting the health of recyclers living in this area.^31^ This means that they may face adverse health risks if they continue to work in e-waste sites without proper waste handling and protection. In addition, the most acute medical needs of these workers remain unassessed.

Barangay Payatas is just one of the many e-waste dismantling sites in the country that discharge e-waste pollutants.^32^ Continuous exposure to different gaseous chemicals released from e-waste may lead to an accumulative biological effect, which is a time-dependent event.^31^ Furthermore, those with a history of working with e-waste had a significant association with increased frequency of the number of micronucleated binucleate cells (MNed BNC), compared to subjects with no history of working with e-waste.^33^

The associations revealed that the length of exposure to e-waste material is a very important contributor to having a greater number of micronuclei. E-wastes contain a range of chemicals that could be released in the sites or environment during the recycling of e-waste as workers use manual techniques in dismantling e-wastes materials.^42^ In addition, they do not have protective gear to mitigate exposures and do not follow prescribed procedures on the use of personal protection equipment due to inadequate knowledge. Recyclers working without any protection at these unsupervised and unlicensed e-waste recycling sites could be exposed to contaminants, as demonstrated by the significantly elevated concentrations of hazardous e-waste chemicals and thereby possibly suffer adverse effects to their health.^43,44,45,46^

People living in e-waste recycling sites or working in e-waste recycling have evidence of greater DNA damage than those living in the control towns with increased frequencies of binucleated cells in their peripheral blood.^47,48^ As seen in previous studies, outcomes associated with exposure to e-wastes include changes in thyroid function, changes in cellular expression and function, adverse neonatal outcomes, changes in temperament and behavior, decreased lung function, increase in spontaneous abortions, stillbirths, premature births, and reduced birth weight and length.^34,35,36,37,38^ Although exposure to e-waste is implicated in DNA damage, studies generally do not have the power to exclude other contributory factors.

Furthermore, e-waste workers engaged in either formal or informal occupations can accumulate chemicals through contaminated soil, air, dust, drinking water, among other sources.^50^ Thus, exposure to e-waste pollution is a very complicated process, with many factors that can trigger health issues. Most of the e-chemicals released from electronic wastes accumulate in the environment and further add to possible adverse health effects.^48,50^

Many studies have focused solely on external exposures to hazardous substances, and not the health consequences caused by these exposures.^51^ A comprehensive analysis, not only of exposures, but also of the health consequences caused by exposures, is essential for an effective occupational medical intervention for e-waste workers in order to minimize occupational hazards and improve their health and safety. Of note is the issue of the transport of environmental contamination. Substantial e-waste pollutants are found not only close to e-waste recycling locations, but also spread to the surrounding environment of soil and water.^42,43^

The results of the present study on the effects of e-waste exposure showing elevated micronuclei, binucleated and other types of cell damage are supported by many other similar studies on micronucleus assays where higher frequencies of binucleated cells in e-waste-exposed populations were observed compared to unexposed controls.^26,50^

The effects of simultaneous exposure to many chemicals through involvement in e-waste recycling are not well explored or understood. This knowledge gap is part of a larger problem in areas where other hazardous wastes are present in the environment.^32,43^ Although e-waste contains a unique combination of persistent hazardous compounds, other sources of exposure are difficult to rule out. Working with e-waste is an independent risk factor associated with genotoxic damage when other variables, such as smoking and age, are accounted for. The mechanisms of action of the mixture of chemicals that make up e-waste are not yet completely known, nor are the full range of effects that arise from the combined exposure to many chemical elements and compounds.^21,47^ In addition, contaminants are also a threat to other organisms, especially those occupying high trophic levels due to biomagnification of these chemicals.

The health effects of exposure to e-waste must become a priority of the international community. Informal e-waste recycling has long been known as a source of dangerous environmental pollution, but the health risks it poses to exposed populations are only beginning to be recognized. An international research agenda should be set by experts to increase the body of evidence of the health effects of e-waste exposure, especially in vulnerable populations. Simultaneously, the international health community, academia, policy experts, and non-governmental organizations, in conjunction with national governments, should create policy solutions, educational programs, and interventions to reduce e-waste exposure and its health effects.

The present study had some important strengths and weaknesses. To the best of the researchers' knowledge, this is a novel study on micronucleus evaluation among informal e-waste workers conducted in Payatas, the Philippines using buccal epithelium cells. Several studies have shown that individuals working in the e-waste industry are continually exposed to e-waste chemicals and face health risks and serious illnesses with longer exposures in this occupation.^28,52^ Further studies are needed to examine the myriad health effects on e-waste workers in order to educate these workers to take appropriate measures as they are unaware of health effects from occupational exposures.^10,26,51^ The present study validated the use of the micronucleus assay as a less invasive and simple method for human biomonitoring among workers exposed to e-waste. However, the absence of longitudinal studies and small sample size are additional limitations of the present study.

## Conclusions

E-waste workers face adverse health risks due to exposure to genotoxins from e-wastes. Micronucleus assays can be used to monitor e-waste workers for genotoxic damage. Further studies are needed to examine relative risks and design strategies to prevent future genotoxic damage in this population as they were observed to have a higher frequency of micronucleated cells and nuclear abnormalities, given the fact that they are susceptible to health related issues such as cancer. The micronucleus assay is now a leading field of interest in mutation research as the prevalent biomarker of chromosomal defects induced by genotoxins in the surroundings as well as in the environment. The present study shows that the micronucleus assay using buccal epithelium can be used for human biomonitoring to evaluate the DNA damage due to e-waste exposure. The mechanism of studying micronuclei using molecular probes and genetically engineered cells can provide a better understanding of gene interactions that would help improve chemical testing, biomonitoring, and prediction of the effects of common genotoxic agents. Based on the current findings, there is an urgent need for a concerted effort by authorities to ensure proper disposal, knowledge, management and enforcement of guidelines, polices and standards in order to address environmental and health hazards posed by exposure to e-wastes. Finally, further studies are needed to understand the role of epigenetic factors in micronucleus formation and abnormalities in human buccal epithelium cells.
